# Effects of olfaction on sexual function, anxiety, depression, and quality of life in men and women with smell disorder: a cross-sectional controlled study

**DOI:** 10.55730/1300-0144.6081

**Published:** 2025-07-13

**Authors:** Esin ÖZLEM ATMIŞ, Eser SAĞALTICI, Hasan GÖKÇAY, Eren YILMAZ, Aytuğ ALTUNDAĞ

**Affiliations:** 1 Department of Otorhinolaryngology, Fulya Hospital, Acıbadem Healthcare Group, İstanbul, Turkiye; 2 Department of Psychiatry, Antalya City Hospital, University of Health Sciences, Antalya, Turkiye; 3 Department of Psychiatry, Şarkışla State Hospital, Sivas, Turkiye

**Keywords:** Anosmia, olfaction, sexual function

## Abstract

**Background/aim:**

Olfaction is necessary for healthy sexual intercourse. Better smell has been associated with a more positive sexual experience.

**Materials and methods:**

In this cross-sectional study, 40 patients diagnosed with a smell disorder and 60 healthy volunteers were included. The participants were divided into 2 groups according to sex. The female group had 19 patients and 25 female controls. The male group had 21 patients and 35 male controls. Sniffin’ Sticks tests, the World Health Organization quality of life assessment (WHOQOL), and hospital anxiety and depression scale (HADS) were applied to all participants. In addition, the international index of erectile function (IIEF) was applied to male participants, and the female sexual function index (FSFI) to female participants. The relationship between the Sniffin’ Sticks test and the WHOQOL, FSFI, IIEF, and HADS scores in both groups was analyzed statistically.

**Results:**

Hyposmic men scored significantly lower on all IIEF subscales (p < 0.001), while hyposmic women scored significantly lower on all FSFI subscales (p < 0.001).

Multivariate logistic regression analysis in men showed 4.9 times lower overall satisfaction (95% CI 1.8–10.1) in hyposmic versus normosmic patients (p = 0.003). Satisfaction was 2.7 times lower (95% CI 1.5–4.8) in hyposmic women compared to normosmic women (p = 0.001).

**Conclusions:**

Both IIEF and FSFI were significantly lower in the hyposmic group. This supports previous research showing that decreased olfactory function is associated with decreased sexual function.

## 1. Introduction

Olfaction is crucial for human sexual behavior [1–3]. Olfaction also plays an important role in reproductive behaviors such as choosing a partner and inbreeding avoidance [4]. Olfactory dysfunction is observed in 12–20% of the population, including anosmia (3.2–3.6%) and hyposmia (9.2–18%) [5,6]. Its prevalence increases with age, reaching up to 62.5% among individuals aged 80–97 years old in a US population-based study [7]. Interestingly, anosmic patients are generally unaware of their inability to smell, and their subjective anosmia assessments are generally higher than their objective assessments. This is important in the context of the clinical importance of objective odor assessment [5]. Although the sense of smell is not given enough importance, it is necessary for sexual intercourse. The sense of smell affects sexual communication [8]. The odors of the secretions in the axillary and genital areas act as chemical signals, stimulating the hypothalamus and causing some hormonal changes [9, 10]. This leads to healthy sexual arousal and desire. Better smell has been associated with a more positive sexual experience. It has also been reported that individuals with high olfactory sensitivity are more likely to enjoy sex and have a higher frequency of orgasm [11]. There are some clinical studies arguing that men have higher sexual desire and increased sexual experience with higher olfactory sensitivity [12]. Bendas et al. [11] showed that sexual pleasure and orgasm frequency increase olfactory sensitivity. In addition, the quality of life of people with olfactory disorders tends to decrease, especially in social interactions and sexual relations [12].

Olfactory function is recognized as an important factor in human sexual behavior and overall quality of life. While prior research suggests that reduced olfactory sensitivity may negatively influence sexual desire, satisfaction, and social interactions, the extent and nature of these effects remain incompletely understood. This study aimed to examine the relationship between olfactory dysfunction, sexual function, and quality of life, considering potential sex differences. We hypothesized that impaired olfaction is associated with lower sexual satisfaction and decreased quality of life, and that sexual function may mediate this relationship differently in men and women. By addressing these questions, we aimed to provide a clearer understanding of the broader psychosocial consequences of olfactory disorders.

## 2. Materials and methods

### 2.1. Participants

This cross-sectional study was conducted between 1 June and 1 September 2021 in the Department of Ear, Nose, and Throat of Fulya and Taksim Acıbadem Hospitals. The participants were 40 patients who were diagnosed with a smell disorder and 60 healthy volunteers. They were all married, between 18 and 55 years old, and having regular sex with their partner. The researchers recruited healthy control subjects partly from their social environment. Control subjects were not from the same family as the patients and were frequency matched to cases by age (±1 year) and sex to reduce potential confounding effects; education level was also recorded and considered in the analyses. The exclusion criteria in both groups were congenital anomalies (isolated congenital anosmia or Kallmann’s syndrome), nasal septum deviation, current upper respiratory tract infection (URTI), sinonasal disease, pregnancy, lactation, significant medical and/or psychiatric pathologies such as schizophrenia, manic-depressive psychosis, dementia, and behavioral disorders with social withdrawal or suicidal risk. Psychiatric disorders were assessed through clinical interviews and review of medical records by psychiatric specialist, in accordance with the criteria outlined in the Diagnostic and Statistical Manual of Mental Disorders, Fifth Edition (DSM-5). The study protocol was approved by the Medical Research Ethics Committee of Acıbadem University Medical Faculty (2021/13 numbered Atadek meeting, approval number 2021-13/03, dated 8 July 2021). The study complied with the Declaration of Helsinki. Written informed consent was obtained from all participants. Any missing data were minimal and handled using pairwise deletion during statistical analysis to preserve available information without introducing bias.

### 2.2. Study design

Subjects were informed of the goals and protocol of the study, and all eligible subjects were asked individually if they wanted to participate. Before enrollment, the general procedure and requirements were explained and written informed consent was obtained. The participants included in the study were divided into 2 groups according to sex. The female group had 19 patients and 25 female controls. The male group had 21 patients and 35 male controls. Sniffin’ Sticks tests, the World Health Organization quality of life assessment-short form (WHOQOL-BREF-TR) and the hospital anxiety and depression scale (HADS) were applied to all participants. In addition, the international index of erectile function (IIEF) was applied to male participants and the female sexual function index (FSFI) to female participants. The relationship between the Sniffin’ Sticks test and WHOQOL-BREF-TR, FSFI, IIEF, and HADS scores in both groups was analyzed statistically.

### 2.3. Data collection

Olfactory performance assessment. The psychophysical testing of olfactory function was performed using the validated Sniffin’ Sticks test (Burghart, Wedel, Germany), for which odorants were presented in commercially available felt-tip pens [13, 14]. First, the pen cap was removed by the experimenter for approximately 3 s for odor presentation. The tip of the pen was placed about 1–2 cm in front of the nostrils. The test consisted of 1 threshold and 2 suprathreshold subtests, namely a test for thresholds of phenylethylalcohol (PEA), a test for odor discrimination (16 triplets with 2 different odors), and a test for odor identification (16 common odors, presented in a 4-alternative, forced-choice procedure). The maximum score of each subtest was 16, resulting in a maximum composite score of 48 for threshold, discrimination, and identification (TDI) [15]. TDI scores of more than 30.3 indicate normosmia, with a cutoff between functional anosmia and hyposmia at 16.5 [16].

### 2.4. Questionnaires

The demographic information of participants was collected by a questionnaire that asked questions about sex, age, education level, job status, and marriage.

The FSFI, IIEF, HADS, and WHOQOL-BREF-TR were used to assess sexual function, psychological symptoms, and quality of life. The FSFI is a 19-item questionnaire evaluating female sexual function across 6 domains, with lower scores indicating greater dysfunction [17–19]; a total score below 26.55 suggests risk of female sexual dysfunction [18]. The Turkish version of the FSFI has been validated by Oksuz et al. [19]. The IIEF evaluates male sexual function in 5 domains, with higher scores indicating better function [20,21], and the Turkish version has also been validated. HADS is a 14-item self-rating scale assessing anxiety and depression symptoms [22,23], with validated reliability in Turkish [23]. WHOQOL-BREF-TR measures quality of life across 4 domains: physical, psychological, social relationships, and environment [24,25], and its Turkish version has established validity and reliability [25].

### 2.5. Statistical analysis

Statistical analysis was performed using SPSS software (version 25.0, SPSS Inc., Chicago, IL, USA). If continuous variables were normally distributed (p > 0.05 for a Kolmogorov-Smirnov test or Shapira-Wilk with n < 30), they were described as the mean ± standard deviation. If continuous variables were not normally distributed, they were described as the median. The continuous variables were compared by using Student’s t-test or Mann-Whitney U test depending on parametric or nonparametric values, respectively. The categorical variables between the groups were analyzed using the Chi-square test or Fisher’s exact test. Variables with a p-value less than 0.1 in univariate analyses were further evaluated using multiple logistic regression. Multiple testing corrections were not applied, as analyses were considered exploratory. The significance level was set at p < 0.05.

A posthoc analysis using IIEF erectile function scores with approximate standard deviations derived from reported minimum and maximum values yielded an effect size (Cohen’s d) of 1.13 and a posthoc power of approximately 98%, indicating the study was well powered.

## 3. Results

The mean age of the 100 participants was 36.0 ± 9.5 (minimum = 19, maximum = 64) years. The TDI of the patient group was below 30.3, indicating hyposmia. TDI results of the control group were above 30.3, indicating normosmia. The mean age was 36.7 ± 8.4 years in the hyposmia group and 35.6 ± 10.1 years in the normosmia group. There was no significant difference between the 2 groups in terms of age (p = 0.552) and education level (p = 0.899) (Table 1).

The sexual function of the participants were analyzed according to sex. Among the 56 males, 21 were hyposmic and 35 were normosmic. Among the 44 females, 19 were hyposmic and 25 were normosmic (Table 1).

There was no significant difference in anxiety and depression scores in hyposmia and normosmia groups within each sex. All 5 IIEF subscales in men (erectile function, orgasm, desire, sexual satisfaction, and overall satisfaction scores) were significantly lower in the hyposmic group than in the normosmic group (p < 0.001 for each one), as was the total IIEF score. All 6 FSFI subscales in women (satisfaction, desire, arousal, lubrication, orgasm, and pain scores) were significantly lower in the hyposmic group than in the normosmic group (p < 0.001 for each one), as was the total FSFI score. The results are summarized in Table 2.

As a result of multivariate logistic regression analysis with IIEF subscales in men, overall satisfaction was found to be 4.9 (95% CI 1.8–10.1) times lower in hyposmic than normosmic participants (Table 3).

As a result of multivariate logistic regression analysis with FSFI subscales in women, satisfaction was found to be 2.7 (95% CI 1.5–4.8) times lower in hyposmic than normosmic participants (Table 4).

There was no difference in anxiety (p = 0.380) and depression (p = 0.555) scores between normosmic (n = 60) and hyposmic (n = 40) groups. The scores from the WHOQOL-BREF-TR were significantly lower in the hyposmic group compared to the normosmic group for national environment domain (p < 0.001), physical health (p = 0.002), psychological health (p < 0.001), social relationships (p < 0.001), and environment (p < 0.001) scales. The results are summarized in Table 5.

As a result of the multivariate logistic regression analysis for the HADS and WHOQOL-BREF-TR subscales in the whole sample, the social relationship levels in the hyposmia group were 1.8 (95% CI 1.4–2.5) times lower than in the normosmia group (Table 6).

## 4. Discussion

Recent studies have associated olfactory dysfunction with diminished social functioning and smaller social networks [26]. Moreover, olfaction appears to influence sexual experiences, as individuals with olfactory disorders may encounter disruptions in their sexual lives, suggesting a broader impact on intimate relationships and social interactions [27]. While there is growing recognition of the significance of scent in sexual behavior, further research is necessary to fully understand its role. Additionally, olfactory deficits have been linked to alterations in mental health and overall quality of life, emphasizing the complex interplay between smell, sexuality, and well-being [28]. In line with these findings, our study indicates that olfactory dysfunction may negatively affect both sexual desire and quality of life, reinforcing the clinical relevance of evaluating olfactory function in patients presenting with sexual difficulties.

The sense of smell plays a crucial role in mammalian sexual behavior, modulating both social communication and reproductive interactions [29]. Olfactory cues, including androgen-like odors, can trigger sexual arousal and hypothalamic activation, highlighting a biological mechanism by which scent influences sexual behavior [30]. Clinical evidence suggests that men with higher olfactory sensitivity have greater sexual desire, further supporting the link between smell and sexual function. Consequently, impairment of olfactory function is likely to negatively impact sexual experiences. Patients with dysosmia often report lower quality of life, affecting social and sexual interactions [12]. Surveys indicate that over 50% of individuals with chemosensory disorders experience adverse effects on sexual behavior, particularly men, with depressed mood exacerbating reduced sexual desire [31]. Additionally, Bendas et al. [11] reported that higher olfactory sensitivity was associated with greater sexual pleasure and, in women, more frequent orgasms during intercourse. Our findings align with the literature. Participants with normosmia scored significantly higher on sexual function scales compared to the hyposmia group, particularly in overall satisfaction, emphasizing the functional importance of olfaction in sexual life.

Olfactory disorders appear to negatively impact sexual experiences, social interactions, and overall quality of life, with better olfactory function correlating with enhanced sexual and social functioning [12]. Previous work suggests that these negative effects may stem from daily life limitations imposed by olfactory deficits, such as reduced taste perception and inability to detect potentially hazardous odors like smoke or poison [12]. Consistent with our findings, individuals with olfactory impairment had lower scores across multiple domains of quality of life, indicating decreased social engagement, deteriorating physical and psychological health, and impaired sexual experiences. These observations highlight the clinical importance of evaluating olfactory function, as it may provide insights into patients’ overall well-being and functioning.

Chemosensory dysfunction has been associated with reduced quality of life and increased levels of anxiety and depression. Previous studies have reported that individuals with olfactory impairment may experience social withdrawal, partly due to concerns about personal hygiene that could contribute to heightened anxiety and depressive symptoms [32]. Additionally, neurobiological mechanisms have been proposed, suggesting that olfactory loss may affect emotional processing through reduced amygdala and limbic system activation [12]. Our study did not find significant differences in anxiety or depression levels between individuals with and without olfactory dysfunction. This contrasts with previous studies reporting more mood disturbances in olfactory impairment [32]. One possible explanation is that the severity and duration of olfactory loss, as well as compensatory behavioral or social adaptations, may modulate the psychological impact. Additionally, individual differences in emotional resilience and support networks could buffer against mood alterations, suggesting that olfactory dysfunction does not uniformly translate to anxiety or depression. Further research is needed to clarify the specific conditions under which olfactory deficits affect mental health.

This study has several limitations. The relatively small sample size and single-center design may limit the generalizability of the findings. Additionally, the cross-sectional nature of the study precludes any causal inferences regarding the relationship between olfactory dysfunction, social functioning, and sexual health. Future research with larger, multicenter, and longitudinal designs is warranted to confirm these findings and clarify the temporal and causal relationships.

In conclusion, our findings underscore the importance of olfactory function in overall well-being and social engagement. Clinicians should consider evaluating olfactory abilities as part of routine assessments, particularly in patients presenting with sexual dysfunction or reduced quality of life. Identifying olfactory impairment early may help guide interventions, provide explanations for subtle social or sexual difficulties, and inform tailored counseling or rehabilitation strategies. Integrating olfactory assessment into clinical practice can therefore enhance patient-centered care and improve holistic health outcomes.

## Figures and Tables

**Table 1:** Demographic characteristics of participants.

Variables	Total (n = 100)	Hyposmi (n = 40)	Normosmi (n = 60)	p
Age, median (Mean±SD), years	36.0 ± 9.5	36.7 ± 8.4	35.6 ± 10.1	0.552
Sex, n (%)				0.356
Male	*56 (56)*	21 (52.5)	35 (58.3)	
Female	*44 (44)*	19 (47.5)	25 (41.7))	
Education, n (%)				0.899
High school	33 (33)	13 (32.5)	20 (33.3)	
University and above	67 (67)	27 (67.5)	40 (66.7)	

**Table 2:** Sexual function indices and HADS scores according to gender.

		Male		
	Total median (min–max)	Hyposmi median (min–max)	Normosmi median (min–max)	p
**IIEF**	**(n = 56)**	**(n = 21)**	**(n = 35)**	
**Erectile Function**	23 (1–30)	18 (1–28)	26 (1–30)	<0.001
**Orgasm**	8 (0–11)	6 (0–10)	10 (0–11)	<0.001
**Desire**	7 (2–10)	5 (3–10)	8 (2–10)	<0.001
**Sexual Satisfaction**	11 (0–15)	8 (0–14)	12 (0–15)	<0.001
**Overall Satisfaction**	8 (2–10)	4 (2–10)	9 (2–10)	<0.001
**Total**	57 (5–75)	41 (6–72)	67 (5–75)	<0.001
**HADS**				
**Anxiety**	4 (2–10)	5 (2–10)	4 (2–10)	0.986
**Depression**	2 (0–7)	2 (0–7)	3 (0–7)	0.499
		**Female**		
**FSFI**	**(n = 44)**	**(n = 19)**	**(n = 25)**	
**Satisfaction**	4.4 (0–6)	0 (0–6)	5.2 (0–6)	<0.001
**Desire**	3.6 (1.2–6)	2.4 (1.2–6)	4.8 (1.2–6)	<0.001
**Arousal**	3.9 (0–6)	0 (0–6)	4.8 (0–6)	<0.001
**Lubrication**	4.2 (0–6)	0 (0–6)	5.1 (0–6)	<0.001
**Orgasm**	3.6 (0–6)	0 (0–6)	4.8 (0–6)	<0.001
**Pain**	4.8 (0–6)	0 (0–6)	5.6 (0–6)	<0.001
**Total**	24.1 (1.2–36)	4.2 (1.2–36)	29.6 (1.2–36)	<0.001
**HADS**				
**Anxiety**	5(0–11)	5 (2–11)	5 (0–9)	0.163
**Depression**	1(0–9)	2(0–9)	1(0–7)	0.749

HADS; Hospital Anxiety and Depression Scale, IIEF; International Index of Erectile Function, FSFI; Female Sexual Function Index

**Table 3:** Multivariate logistic regression analysis results for IIEF.

	B	p	Odds Ratio	95% C.I.for Odds Ratio	
				Lower	Upper
**IIEF**					
Erectile Function	0.044	0.854	1.04	0.65	1.67
Orgasm	−0.291	0.513	0.75	0.31	1.79
Desire	−0.455	0.267	0.63	0.28	1.42
Sexual Satisfaction	−0.311	0.492	0.73	0.30	1.78
Overall Satisfaction	1.600	**0.003**	**4.95**	**1.74**	**10.1**
Constant	−2.562	0.042	0.077		

IIEF; International Index of Erectile Function

**Table 4:** Multivariate logistic regression analysis results for FSFI.

	B	p	Odds Ratio	95% C.I.for Odds Ratio	
				Lower	Upper
**FSFI**					
Satisfaction	0.991	**0.001**	**2.69**	**1.51**	**4.82**
Desire	−0.342	0.606	0.71	0.19	2.60
Arousal	−0.165	0.867	0.85	0.12	5.84
Lubrication	0.769	0.573	2.16	0.14	31.33
Orgasm	0.130	0.865	1.14	0.25	5.11
Pain	−1.309	0.238	0.27	0.03	2.38
Constant	−2.220	0.149	0.109		

FSFI; Female Sexual Function Index

**Table 5:** Comparison of HADS and WHOQOL-BREF-TR scores between hyposmia and normosmia groups.

	Total (n = 100)	Hyposmi (n = 40)	Normosmi (n = 60)	p
	Median (Min–Max)	Median (Min–Max)	Median (Min–Max)	
**Anxiety**	5 (0–11)	5 (2–11)	5 (0–10)	0.380
**Depression**	2 (0–9)	2 (0–9)	2 (0–7)	0.555
**National environment domain**	9 (2–10)	7 (3–10)	9 (2–10)	<0.001
**Physical health**	31 (12–35)	26 (12–35)	31 (15–35)	0.002
**Psychological health**	26 (9–30)	22 (9–30)	27 (12–30)	<0.001
**Social relationships**	11 (3–15)	9 (3–15)	13 (7–15)	<0.001
**Environment**	35 (9–40)	30 (9–40)	37 (19–40)	<0.001

WHOQOL-BREF-TR; The World Health Organization Quality of Life Assessment – Short Form, HADS; Hospital Anxiety and Depression Scale

**Table 6:** Multivariate logistic regression analysis results for HADS and WHOQOL-BREF-TR.

	B	p	Odds Ratio	95% C.I.for Odds Ratio	
				Lower	Upper
**National environment domain**	−0.010	0.969	0.990	0.594	1.649
**Physical health**	−0.194	0.104	0.824	0.653	1.040
**Psychological health**	0.065	0.609	1.068	0.831	1.371
**Social relationships**	0.544	**0.001**	**1.773**	**1.360**	**2.556**
**Environment**	0.045	0.554	1.046	0.902	1.212
**National environment domain**	−0.148	0.377	0.863	0.622	1.197
**Anxiety**	0.119	0.437	1.126	0.835	1.520
**Depression**	−2.679	0.118	0.069		
**Constant**	−0.010	0.969	0.990	0.594	1.649

WHOQOL-BREF-TR; The World Health Organization Quality of Life Assessment – Short Form, HADS; Hospital Anxiety and Depression Scale
